# The African digital health student bootcamp: bridging education, workforce, and practice gaps for healthcare innovation in Sub-Saharan Africa

**DOI:** 10.3389/fdgth.2026.1728386

**Published:** 2026-02-04

**Authors:** Tolulope Israel Oni, Rawan Mahmoud Elrayah Hussein, Olutola Vivian Awosiku, Shola Paul Emiade, Oluomachi Joline Osuagwu, Victor Femi-Lawal, Jide Babayeju, Lucy Anne Waweru, Moses Mathenge, Taofeekat Adigun, Loveday Nnabuife, Opeyemi Precious Alalade, Olaniyi Onigbinde

**Affiliations:** 1 Digital Health Africa, Ondo city, Ondo State, Nigeria; 2 Department of Basic Medical Sciences, Kwara State University, Ilorin, Nigeria; 3 Faculty of Dentistry, University of Khartoum, Khartoum, Sudan; 4 Department of Medicine, University of Nigeria Teaching Hospital (UNTH), Owerri, Nigeria; 5 College of Medicine, University of Ibadan, Ibadan, Nigeria

**Keywords:** African students, bootcamp, digital health, empower, healthcare, innovation, youth

## Abstract

**Introduction:**

Africa, despite significant progress in various sectors, continues to face public health issues largely due to factors such as inadequate health system financing mechanisms, lack of stable leadership and governance, shortage of skilled health workforce, and limited access to drugs and technologies. To address these challenges, it is crucial to build the capacity of future professionals, providing them with resources to innovate health solutions. As such, we designed a bootcamp that empowers students, supports collaboration and interdisciplinary learning, provides access to mentors and resources, and fosters the development and application of practical digital health concepts for African university students.

**Methods:**

The eight-week virtual bootcamp brought together diverse university students from across Africa, regardless of field or level of knowledge. They received mentorship and workshop sessions on the basics of digital health concepts, such as Basic Introduction to Digital Health, Healthcare Data and Analytics, etc., to develop health solutions. They were divided into teams of five, assigned mentors for collaborative projects, and pitched them. Winners and outstanding participants were awarded cash prizes. Pre- and post-boot camp surveys were conducted to assess changes in participants’ knowledge and skills.

**Results:**

A total of 47 participants aged 18–35 years completed the survey, with an average age of 24, comprising 24 females (51%) and 23 males (49%). They were from 13 countries across the continent, with the majority, 20 (42%), from Nigeria, Tanzania, and Ethiopia, each with 5 (11%), Kenya 4 (9%), Zambia, and Ghana, with 3 (6%) each. The remaining seven countries shared a total of 15%. Before the bootcamp, most participants (45; 94.83%) were somewhat unfamiliar with digital health concepts. After the bootcamp, (40; 82.93%) of participants reported a significant improvement in their knowledge, skills, and experience with digital health concepts.

**Discussion:**

The Africa Digital Health Student Bootcamp 2023 (ADHSB'23) empowered participants with digital health skills and knowledge. Participants demonstrated significant improvements in their familiarity with digital health concepts. Therefore, it is necessary to showcase this creativity and innovation to stakeholders such as digital health organizations, universities, policymakers, and the general public to raise awareness and recognition of the program.

## Introduction

Africa, despite making significant strides in various sectors, grapples with persistent public health challenges, which could be largely attributed to several factors such as inadequate health system financing mechanisms, lack of stable leadership and governance, shortage of skilled health workforce, limited access to drugs and technologies, and weak health information systems (1). As the continent faces a myriad of health challenges, ranging from communicable to non-communicable diseases (2), the young people of the continent must be adequately empowered to face these challenges. In 2021, the rate at which individuals use the internet in Africa increased from 4% in 2008 to 33%, identifying this strategic opportunity amidst the pressing health challenges in the African region, the World Health Organization (WHO) directed each Member States to effectively implement the global digital health strategy with a maximum sustainability model (3). In light of these directives and existing challenges, the Africa Center for Disease Control and Prevention (AfricaCDC) rolled out the Digital Transformation Strategy (DTS), which serves as a significant step towards improving public health outcomes on the continent through the use of technology. It is believed that innovative approaches can play a transformative role in empowering people, strengthening health systems, and supporting sustainable development (4). One of the strategies highlighted by AfricaCDC is developing workforce capacity by equipping them with the knowledge, skills, and competencies to develop, implement, and sustain digital health initiatives. Aside from that, it also directed each Member State to create a national digital health strategy that mirrors the AfricaCDC DTS, in which workforce capacity building is an essential aspect in each nation's strategy.

As the continent continues to experience a demographic shift towards a younger population with nearly 60% of its population under 25 years of age, it is also reported that by 2030, these young people will make up more than 40% of the global youth population (5). In light of these opportunities, it is evident that harnessing the energy and commitment of this burgeoning youth, who represent 60% of Africa's population, is not a matter of choice but an imperative for sustainable development (5, 6).

Despite these opportunities, the African young people are still overlooked and often underrepresented when it comes to making key decisions in the development space. This represents a huge missed opportunity as it negates the standpoint of the United Nations Convention on the Rights of the Child (7). Aside from this, most African young people often face existential circumstances, including low literacy levels, unemployment, and other cultural and socioeconomic factors (8), making it difficult for youth to ideally contribute to sustainable developments, particularly good health and well-being.

In recent times, emphasis has been shifted to building African young people's capacity, bridging the literacy gap, and linking them with resources that will enable them to fully harness their creative and innovative power and potentially contribute to and transform the continent's sustainable development (9). This is because young African people can play a major role in shaping the innovative approach to healthcare because of their diverse backgrounds, different ideas, and solutions that foster change for better health outcomes and aim to achieve universal health coverage for all (5).

Moreover, young people are instrumental to health innovation because they possess a creative mindset and fresh perspective, are technology-savvy, and have the community to escalate their solutions (10, 11). However, youth innovations in Africa face several challenges, such as a lack of resources, insufficient funding, inadequate mentorship, low literacy levels, and inadequate skills, which hinder these young people's contribution to solutions on the continent (5). Consequently, it is crucial to build the capacity of students who are future professionals to be able to innovate health solutions, providing them with the necessary resources and network needed to propel innovation in the field of digital health, leading to digital health literacy (12).

Platforms such as bootcamps that support collaboration, interdisciplinary learning, and the development and application of practical digital health concepts are potential solutions to providing capacity-building opportunities (13). Several studies (11, 12, 14–16) conducted across various overlapping age ranges of 18–24 or 18–35 years have highlighted the importance of bootcamps in empowering and building the capacity of this demographic with digital health skills.

They are often designed to bring diverse individuals with different backgrounds and skill sets together, conducted over a period of time, and drive interdisciplinary collaboration on innovative projects. Bootcamps also supplement students' skills because they differ from traditional learning models and expose them to practical skills and real-life application of acquired skills (13). Such a platform would enable African students to develop new solutions or modify existing ones to Africa's specific healthcare concerns. In the absence of such a platform, prospective digital health professionals (students and young people) may find it challenging to bridge the gap between theory and practice, limiting their impact on the healthcare sector.

The African Digital Health Student Bootcamp 2023 (ADHSB'23) aimed to empower young people with theoretical and practical knowledge to innovate health solutions tailored to the African context. This paper describes the process of organizing and implementing the bootcamp, highlights the innovative solutions developed by the students, and discusses the role of ADHSB'23 in empowering Africa's young people for healthcare innovation and bridging education, workforce, collaboration, and practice gaps in Sub-Saharan Africa.

## Methods

### Study design

ADHSB'23 was designed as an eight-week virtual training program incorporating mentorship and workshop sessions each week, culminating in an innovation challenge after completing their ideation. It empowers 50 students with the requisite knowledge, skills, and network to spearhead innovation and catalyze healthcare advancement through digital technology across Africa. The participants were given instructions qualitatively, allowing for real-time feedback, questions, and responses, but the results were assessed from the pre- and post-surveys. In addition, the bootcamp's qualitative instruction and real-time feedback help mentors understand students' experiences and perspectives about digital health, encouraging them to think creatively and develop innovative approaches and solutions to address global health issues. In addition to learning theoretical and practical skills, bootcamps usually exhibit a time-bound format where the participants gather together to learn experientially under their assigned mentors with the attempts to complete a project interesting to them and solve a potential issue (13). The bootcamp was developed with the mindset of empowering the next generation of health professionals, empowering them with the required capacity to ideate for health using a youth-focused approach, highlighting their importance in meaningfully participating in collaborating, mentorship, and innovating for problem-solving (14). In the same vein, the successful applicants aged 18–35 were also considered based on alignment with career goals, academic background, and potential for impact. This bootcamp also infuses the critical thinking approach to successfully apply the digital literacy skills that have been learned during the mentorship and workshop to practically and contextually develop fundable innovative ideas that are peculiar to the African population. Additionally, the design thinking approach to mentorship sessions guides the students through the stages of ideating, including defining a problem, ethical considerations, ideating culturally sensitive solutions, prototyping, and testing.

### Planning and implementation

This bootcamp was organized and implemented by Digital Health Africa (DHA). DHA is a youth-led NGO established in 2023 and registered in Rwanda with over 40 young professionals driving the mission across the African Continent. It aims to tackle the arduous task of creating a centralized resource that pools together relevant information on activities, trends, gaps, challenges, and opportunities that are occurring within the digital health landscape across the African continent to inform better decision-making, which would in turn facilitate the development of better health systems.

DHA also promotes youth participation in digital health through the African Digital Health Student Network (ADHSN), one of the largest communities of young people researching and innovating in healthcare. The ADHSN boasts of 575 student members across 50 universities in Africa. This network focuses solely on digital health and on enhancing members' capacity in digital health concepts and practices to innovate for health on the continent, unlike other existing communities or networks that focus on Antimicrobial Resistance (AMR), public health, and medical students' associations. ADHSN's sole focus on digital health currently makes it the largest community of undergraduates across Africa.

#### Aim

ADHSB's overarching theme is shaping the future of African health, and this flagship program aims to empower 50 students with the requisite knowledge, skills, and network essential to innovate and catalyze healthcare advancement through digital technology across Africa. This would be achieved through the following specific objectives.

### Objectives

Engage experienced professionals and experts in digital health to serve as instructors, mentors, and speakers.
Develop a comprehensive curriculum that covers key topics in digital health, including telemedicine, health information systems, mobile health, and health data analytics.
Provide access and expose participants to demos on digital health tools, software, and platforms to facilitate hands-on learning and practical applications.
Forge partnerships with universities, healthcare organizations, incubators and industry stakeholders to ensure access to resources, promote collaboration, and enhance internship opportunities for participants.
Empower participants in identifying healthcare challenges in their community and building uniquely tailored tech-driven solutions.

### Metrics and targets

It is crucial to measure progress, evaluate performance, ensure accountability, and improve the quality and impact of ADHSB to adapt the lessons learned in future bootcamps. For this reason, a structured approach was employed to track metrics and targets, divided into short-, medium-, and long-term targets/impact.

*Short-term metrics and targets (immediately after the bootcamp: 0–3 months)*

Skill Acquisition
  Metric: Improvement in participants’ digital health skills and knowledge before and after the bootcamp using pre- and post-assessment.
  Target: Demonstrated increase in knowledge scores across all participants
Project Completion
  Metric: Number of projects initiated during the bootcamp and the percentage of projects completed successfully by the participants.
  Target:10 Projects from the Bootcamp (5 members per team)
Innovation
  Metric: Number of innovative ideas, solutions, or prototypes developed by the participants during the bootcamp, indicating their ability to think critically and innovate in the digital health domain.
  Target: At least five innovative ideas by the end of the bootcamp.

*Medium-term metrics and targets (Within 3–12 months post bootcamp)*

4. Job Placement
  Metric: Percentage of participants who secure internships, employment, or other relevant digital health opportunities.
  Target: 3–4 internship placements within health tech organizations on the continent.
5. Start-up Readiness and Fundability
  Metric: Number of participants or teams whose ideas mature into viable, fundable digital health venture concepts.
  Target: At least three fundable startup ideas after the bootcamp.
6. Industry Impact:
  Metrics: Assess the extent to which the bootcamp participants contribute to the digital health industry by measuring the number of research publications, presentations, or contributions to relevant conferences or academic events.
  Target: 2–3 invitations to speak or present at health or digital health conferences.

*Long-term metrics and targets (After one year or beyond)*

7. Alumni Engagement and Ecosystem Building
  Metric: Track the level of engagement and continued involvement of bootcamp alumni by measuring their participation in alumni events, mentorship programs, or contributions to the digital health community.
  Target: Establish a Digital Health Student Network across five universities in Africa, led by bootcamp alumni.

### Bootcamp implementation

The bootcamp was implemented in three phases. This regimented implementation strategy allowed us to systematically execute each essential component of our work plan and ensure alignment with overall objectives.

#### First phase

##### Mentors/speakers curation and contract signing

We open a call for applications for mentors by highlighting the courses in the bootcamp curriculum that need mentorship and the aim of the bootcamp. We also leveraged partner organisations and individual networks to identify and select mentors who fit the courses for which we didn't receive high-quality applications or for which we aren't convinced of a particular prospective mentor's skills and capabilities. We carefully selected a diverse group of digital health experts in Africa across various endeavors, including Co-founders/CTOs, Product Managers, Program Analysts, Product Designers, Innovation Researchers, Data Scientists and Analysts, Business Developers, etc. After this rigorous mentor selection process, they signed a contract.

##### Recruitment and selection

**Figure 1:**
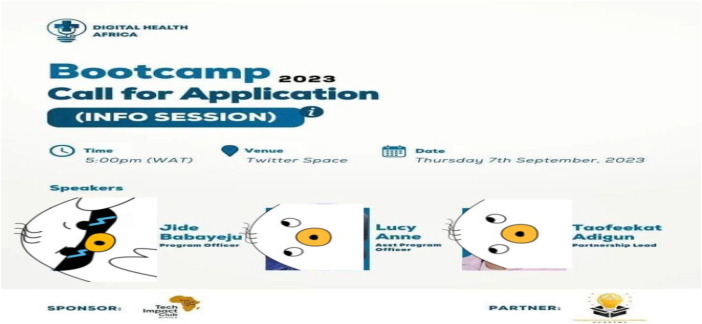
An awareness flyer for the bootcamp information session.

**Figure 2:**
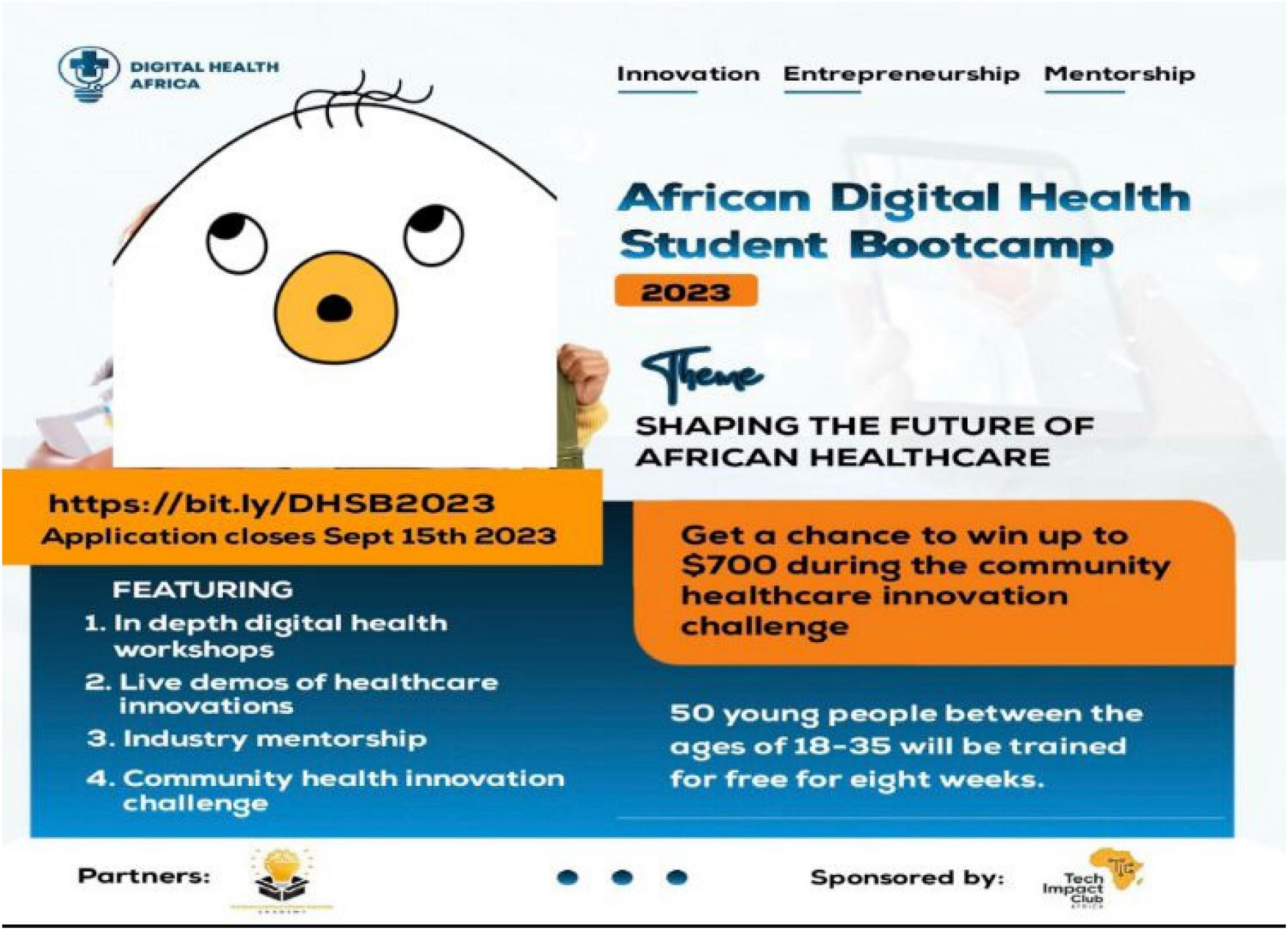
A call for application flyer for the bootcamp.

Before the call for applications was announced, an information session was held on X (formerly called Twitter) to inform people and prospective applicants about the bootcamp objectives, answer questions, and provide clarifications on the program as shown in Figure 1. The recruitment process was conducted through an announcement using an electronic flyer and an open call for applications, which was promoted on various digital health Africa social media platforms, including LinkedIn, WhatsApp, X (formerly Twitter), email newsletters, partner organizations, individuals, and networks as shown in Figure 2. This process ensures a broader reach to diverse students and representation across the continent. The announcement included details about the bootcamp objectives, eligibility criteria, application deadlines, and the application process. This approach incorporated elements of equity and accessibility, giving students from different geographic locations the opportunity to apply. We received a total of 144 applications. A selection committee from Digital Health Africa reviewed applications against predetermined criteria, including academic background (Medicine, IT, Engineering, Computer Science, Allied Health, and a few social sciences), interest in digital health, and potential for impact. At the end of the review process, a diverse group of 50 motivated and talented students was carefully selected, with a gender-balanced ratio.

##### Onboarding

50 selected participants, in addition to 17 student ambassadors from the DHA community, totalling 67 final participants, were notified of their acceptance into the bootcamp and provided with onboarding materials, including program schedules, Google Classroom platform access instructions, and introductory resources on digital health. The onboarding process familiarized participants with the logistics and expectations of the bootcamp, preparing them for a smooth transition into the program. Out of 67 participants who accepted, only 64 participants honoured the invitation to be onboarded on Google Classroom and enrolled in the program.

##### Pre-assessment survey

A pre-assessment survey was developed by a group of experts in accordance with the bootcamp curriculum and administered to participants. It was designed to assess their baseline knowledge, skills, and interests in digital health.

##### Orientation

The orientation session marked the official commencement of the bootcamp and served as an introduction to the program objectives, curriculum, mentors, and key stakeholders. Facilitators from DHA and The Innovation and Design Thinking Academy (TIDA) conducted the orientation session, providing an overview of the bootcamp structure, expectations, and opportunities for engagement. The Senior Technical Officer for Strategic Programmes for the Africa Centre for Disease Control was the keynote speaker. He motivated and inspired the young participants while also challenging them to be bold, innovative, and creative in helping Africa overcome its healthcare challenges.

##### Curriculum delivery

A standard and intensive curriculum that integrated case studies, workshops, lectures, mentorship, and group projects to facilitate the acquisition of digital health knowledge and skills in areas such as design thinking in healthcare, change management in digital health, AI & emerging innovations in healthcare, healthcare data analytics, healthcare entrepreneurship, and product management in healthcare innovations was delivered for seven weeks.

#### Second phase

##### Mentorship

Each week, we held two mentorship sessions and a workshop for the entire cohort. The mentorship sessions focus on the technical aspects of the curriculum, including Basic Introduction to Digital Health, Healthcare Data and Analytics, Digital Health Innovations and Emerging Digital Healthcare Technologies, Design Thinking in Digital Healthcare/Human-Centered Design Thinking, and others. Meanwhile, the workshop session covers a less technical aspect of the program, including Collaboration and Networking skills, Demos from AI/healthtech Companies, Case Studies, and Fireside Chats with digital health startup founders, among others. These intensive sessions ran for four weeks via Zoom, with each lasting approximately 2 h. Each of the digital health experts and mentors taught the students, shared their knowledge based on the curriculum and their areas of expertise, provided insights into the industry landscape, and offered invaluable advice. This mentorship and workshop not only enriched the learning experience but also opened doors for potential collaborations and future opportunities through vital skills in design thinking, context-specific solution design, and effective pitching (15).

##### Practical application to collaborative projects

The participants were divided into 10 teams and encouraged to collaborate on developing innovative digital health solutions or initiatives that address specific healthcare challenges in Africa over the following three weeks. They were appointed a mentor to guide them through this phase. These methods enable a collaborative approach and engagement of team members throughout the process (17).

##### Bootcamp pitches

A final event was organized for participants to showcase their projects and pitch their ideas to a panel of experts and judges. The projects were critically reviewed by the judges using the following criteria: problem identification, problem-solution fit, novelty and creativity, feasibility, sustainability, impact, clarity, presentation, and team collaboration (18).

#### Third phase

##### Post-bootcamp assessment

A post-assessment survey was developed by the group of experts in accordance with the bootcamp curriculum. This was conducted to understand the level of knowledge increase and skills acquired by the participants of the ADHSB. It also evaluates their overall experience, assessing the effectiveness of the curriculum and identifying areas for improvement. During the training, those who were inactive and missed two sessions were removed owing to time zone variances across the continent, as well as personal reasons. As a result, only 54 participants graduated, and only 47 of those completed the survey, with others giving reasons relating to individual and infrastructure concerns for not completing it, representing a response rate of 75%.

##### Skills and competencies developed

The participants' level of knowledge and skills was assessed before and after the boot camp using pre- and post-assessment surveys.

##### Close-out event

The graduation ceremony marked the culmination of the ADHSB, an eight-week virtual program. The ceremony served as a platform to celebrate the achievements of the 54 students who successfully completed the bootcamp, recognizing their dedication, innovation, and contributions to the field of digital health in Africa. Outstanding participants and projects were recognized and awarded based on criteria such as innovation, impact, and teamwork. Awards included the Best 3 Projects, Most Active participant, Most punctual participants, and Overall Best Students (Male/Female). Several participants shared their reflections and testimonials about their bootcamp experience, expressing gratitude for the knowledge gained, the friendships formed, and the opportunities created through the program.

### Data collection and analysis

Data was collected through online surveys. Pre- and post-bootcamp surveys were used to gather quantitative data from participants, including demographic information as well as feedback on their experiences and suggestions for future improvements. To assess the success of the event, session recordings and logs were utilized to track participation and engagement. Data collection and analysis were conducted using Microsoft Excel and SPSS version 27.0.1.0, respectively.

## Result

**Figure 3:**
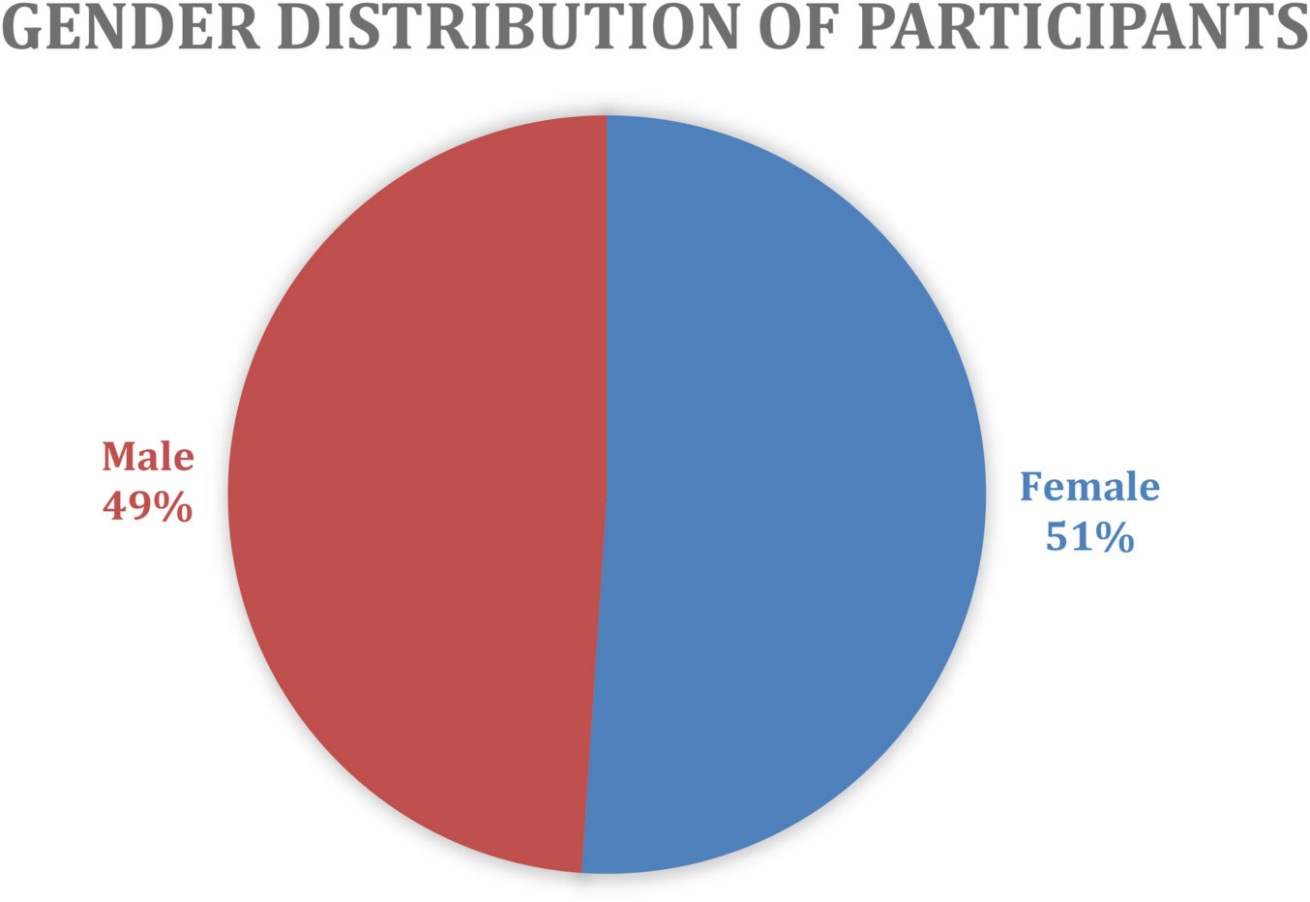
Gender distribution of participants.

**Figure 4:**
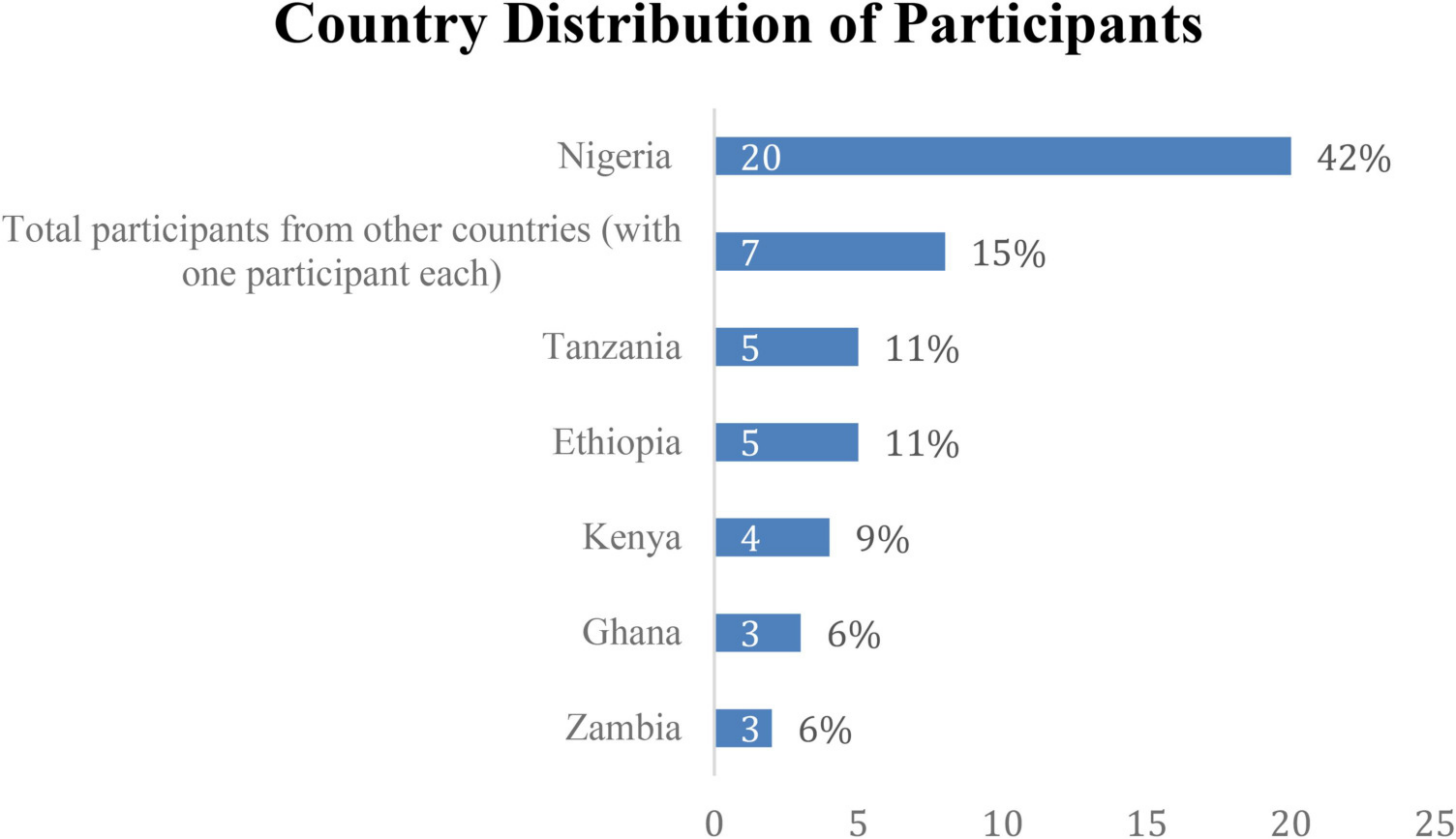
Country distribution of participants.

A total of 64 participants enrolled in the program, but only 47 completed the program and survey, with a mean age of 24 (2.58), as shown in Table 1. Of these, 24 were females (51%), and 23 were males (49%), as shown in Figure 3. The majority (85.1%) of participants were undergraduates, while 14.9% were postgraduate students, as shown in Table 2. Moreover, most participants (21.3%) are in their fourth year of study, followed closely by those in their second and sixth years (17%) each, as shown in Table 3. The participants were from 13 countries across the continent, with the majority (42%) from Nigeria, followed by Tanzania and Ethiopia, each with 11%. (9%) were from Kenya, (6%) from Zambia and Ghana, and the rest of the seven countries shared the same percentage of (15%), as shown in Figure 4. Also, the majority of participants (36.2%) were in medicine and surgery, followed by pharmacy (10.6%), medical microbiology, nursing, and statistics (4.3%), with the remaining participants at 2.1% each, as shown in Table 4.

**Table 1:** ADHSB participants’ Age group.

Age group	Frequency	Percent
19–21	10	21
22–24	22	47
25–27	11	23
28–30	3	7
31 and above	1	2
**Total**	**47**	**100.0**

Mean age = 24 ± 2.58.

**Table 2:** ADHSB participants’ distribution by level of study.

Level of study	Frequency	Percent
Postgraduate	7	14.9
Undergraduate	40	85.1
**Total**	**47**	**100**.**0**

**Table 3:** ADHSB participants’ distribution by year of study.

Year of Study	Frequency	Percent
Year 1	7	15
Year 2	8	17
Year 3	7	15
Year 4	10	21
Year 5	7	15
Year 6	8	17
**Total**	**47**	**100.0**

**Table 4:** ADHSB participants’ distribution by course of study.

Course of study	Frequency	Percent
B.Sc. Health Information Management	1	2.1
Bachelor of Medical Laboratory Sciences	1	2.1
Bachelor of Science in Computer Engineering and Information Technology	1	2.1
Bachelor of science in optometry	1	2.1
Bachelor of Business Administration Honours	1	2.1
Bachelor of Public Health	1	2.1
Biomedical Engineering	1	2.1
Biomedical Laboratory Science	1	2.1
BSc Computer Science and Economics	1	2.1
Health Education	1	2.1
Health Informatics	1	2.1
Health Promotion and Public Health Education	1	2.1
Master of Public Health	1	2.1
Medical Laboratory Sciences	1	2.1
Medical Microbiology	2	4.3
Medical Physiology	1	2.1
Medicine and Surgery	17	36.2
Nursing	2	4.3
Pharmacy	5	10.6
Physiotherapy	1	2.1
Psychology	1	2.1
Public Health Nutrition	1	2.1
Software engineering	1	2.1
Statistics	2	4.3
**Total**	**47**	**100**.**0**

### Participant's familiarity with digital health concepts and levels of improvement

**Figure 5:**
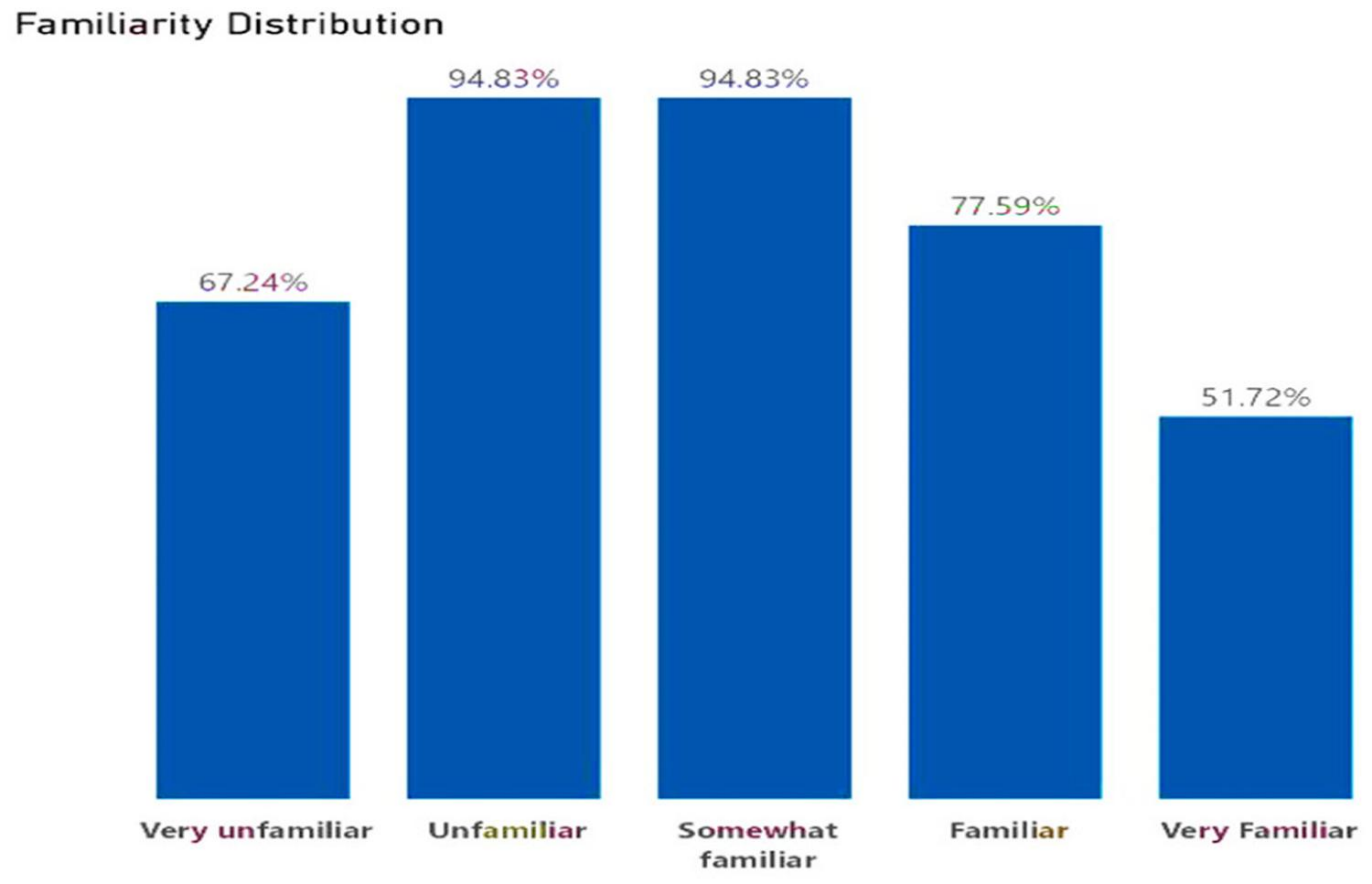
Familiarity distribution with digital health concepts.

**Figure 6:**
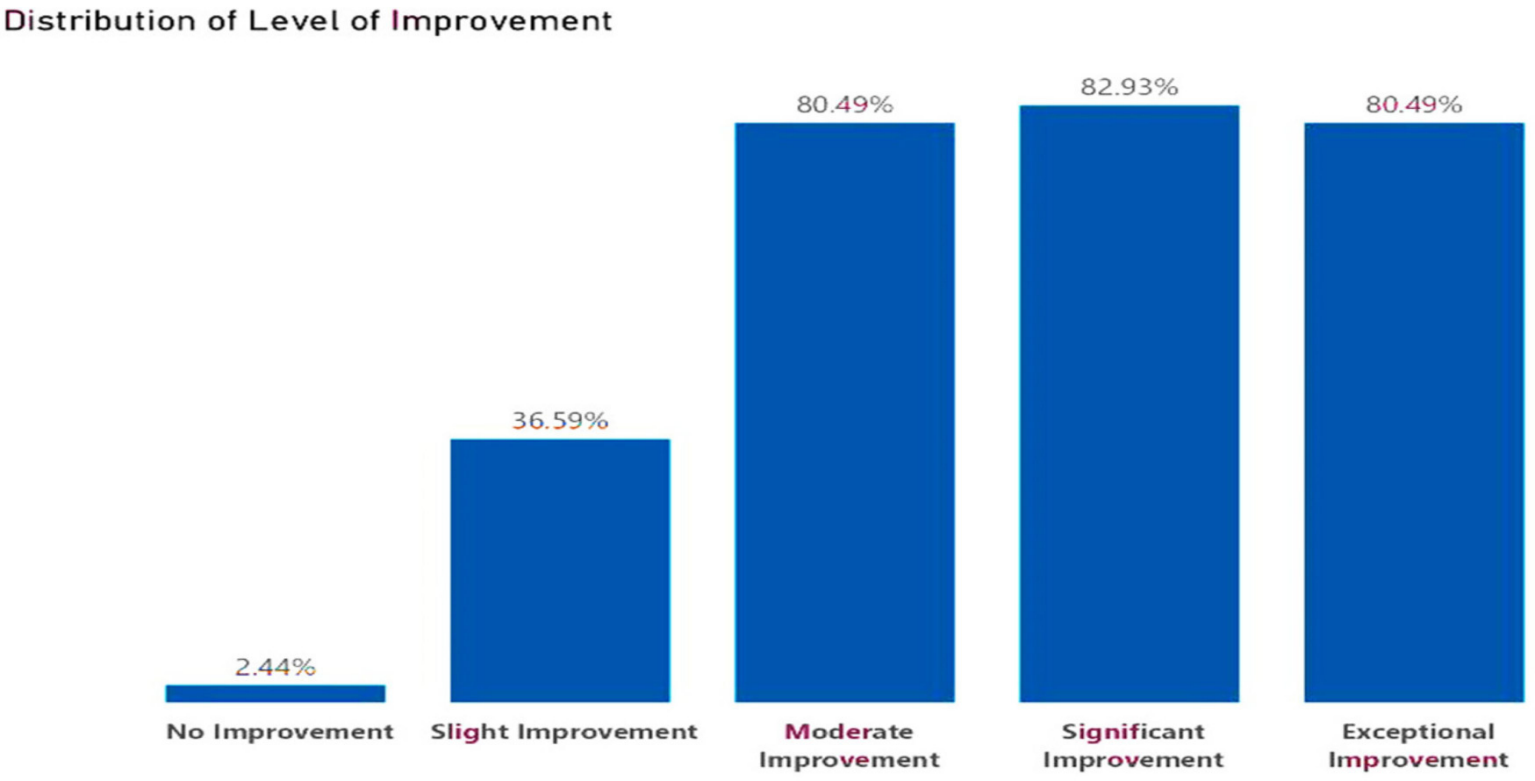
Levels of improvement.

At baseline, the knowledge, skills, and experience with digital health concepts were recorded at 2.63% and this increased by 1.4% post-bootcamp. Before the boot camp, most participants were somewhat unfamiliar with digital health concepts at 94.83%, followed by a group of participants who were unfamiliar with the concepts at 93.10%. Only 48.28% of the participants were very familiar with digital health concepts. After the bootcamp, 82.93% of participants reported an improvement in their knowledge, skills, and experience with digital health concepts, followed by 80.49% of participants who recorded an exceptional improvement. Only 2.44% of participants reported no improvement in digital health concepts, as evidenced in the following charts, in Figures 5 and 6.

### Participants' average familiarity with the modules and level of improvement

**Figure 7:**
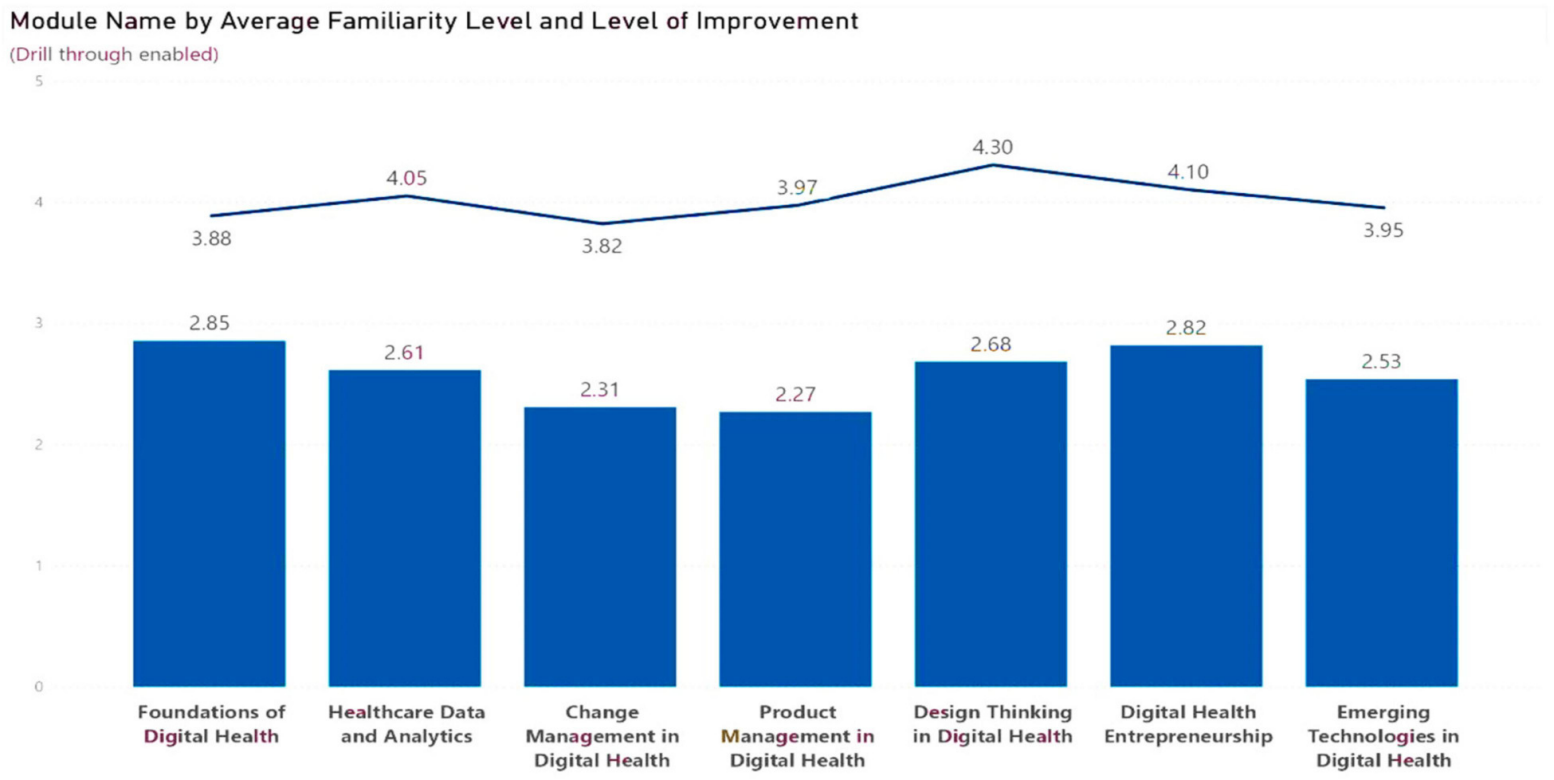
Average familiarity and levels of improvement.

As shown in the chart below in Figure 7, improvements in knowledge were registered across the seven modules that comprised the ADHSB curriculum. The highest degree of improvement was observed in product management in digital health, at 1.7%, followed by design thinking in digital health, at 1.62%. The foundation of digital health noted the least improvement at 1.03. Similarly, participants explained the application of knowledge acquired during the boot camp by sharing the use of the design thinking process, specifically empathy research, ideation, and prototyping, in assessing the barriers to the use of digital health in healthcare and incorporating innovative solutions into patient care. Additionally, the participants expressed that the boot camp will aid their career growth and development.

#### Audiences for proposed solutions

After a successful demo day, the judges released their results, and the winners of the challenge were selected. **Team 8,** which developed an AI platform for the early detection of lung cancer in Africa, emerged as the overall winner of the Community Health Innovation Challenge (CHIC). Team members are DHSB16, DHSB45, DHSB46, DHSB48, and ADHSN11, clinching $700 in the process. **TEAM 3,** with a digital platform that delivers interactive cervical cancer education, emerged as the first runner-up and won $500. In third place is **TEAM 4,** whose project aims to make accurate and credible HPV vaccination information and education accessible to the Nigerian populace through a short-code service system in the local language, and won $300. Table 5 below presents the complete list of ideas generated during the bootcamp and the audience/populations for the proposed solutions.

**Table 5:** Project ideas and the audience/populations for proposed solutions.

Final position	Team ID	Number of members	Theme	Proposal	Digital approaches	Score
1	Team 8	5	Revolutionizing health care challenges through early detection	INNOTECH, an AI platform for early detection of lung cancer in Ghana, Ethiopia and Benin	mHealth/AI	73.5
2	Team 3	6	Simplified health education	EDUHEALTH, A digital platform that delivers interactive cervical cancer education and convenient services at your comfort zone in Nigeria	Web-based technology	72.5
3	Team 4	5	Vaccine reach made easy	VAX-EASY, A platform that makes accurate and credible HPV vaccination information and education accessible to the Nigerian populace through a short-code service system in the local language in Nigeria	SMS-based mHealth platform	72
4	Team 9	6	NA	NA	NA	NA
5	Team 7	5	Your Record.@Your Control.@Your Health.	RICA HEALTH, an online medical health record in Ethiopia	EMR	69.5
6	Team 6	5	Feel and be safe	MySafeSpace is a website designed to offer accessible, reliable, and cost-effective information on sexual, reproductive, and mental health for individuals aged 17–25 in Nigeria	eHealth/AI	68
6	Team 2	6	Every minute counts	AFRIWELL INNOVATIONS is a platform that provides comprehensive medical emergency handling information and transportation via licensed professionals, tailored to the African population in Kenya	mHealth (mobile app)	68
8	Team 1	5	Innovative Diagnostics, Unmatched Efficiency	RADIOVISION, a game-changing digital solution to the challenge of pneumonia diagnosis.	Machine learning technology	67.5
9	Team 5	6	Patient empowerment	MEDSYNC, A comprehensive self-management tool for low health literacy patients with chronic diseases	eHealth	65
10	Team 10	5	CEEH	CEEHEDGE will reduce the mortality rate amongst people diagnosed with hypertension and diabetes in Nigeria and Rwanda	mHealth/Mobile app	56

### Challenges faced by participants

The significant challenges faced by participants included access to reliable internet connectivity and digital devices, which remain a challenge for some participants and limit their ability to engage in virtual learning activities fully, as reported by participants who dropped out. This highlighted the need for improved digital infrastructure and support systems. Furthermore, the varying time zones across the continent and balancing academic commitments with the demands of the bootcamp have been difficult for some participants, leading to time management issues and potential dropouts. Also, making cross-border payments was a significant challenge due to exchange rate charges and the difficulty of finding a suitable platform that supports cross-border transactions, which led to delays in payments to faculty and prize winners.

### Participants' feedback/suggestions for future bootcamps

The participants have a series of structured feedback and suggestions for future bootcamps' success. These suggestions focus on the quality of the bootcamp and its impact, the result of the qualitative response was also tailored to each of the objectives and the impact it made on the lives of these students. Below are the responder ID tags for each feedback and suggestion provided by the participants, as assessed from the surveys.

### On instructors (objective 1)

“The instructors were incredible! Their passion for digital health was amazing, and they were always there to help us. I just hope you will all accept our invitations to connect on LinkedIn.”-A*DHSB25*

“They were Good and Very passionate to us and very encouraging, especially I liked them a lot it was good to learn from them as a Young digital health enthusiast.”-*ADHSB33*

“Very impressive delivery and contents shared by instructors.”-*ADHSB48*

“They all added something significant and impacted knowledge in their sessions.”-*ADHSB26*

### On the curriculum and content delivered to participants (objective 2)

“The session on Design thinking—ideation, prototyping, human-centered design, empathy research, etc., in Digital Health helped me to make adjustments and modifications to the idea I was working on for my organization with regards to blood supply and delivery systems. I realized I had made assumptions on certain things without actually doing empathy research on real human beings/patients to see what their pain points were, so that our digital solution could address *them* with the perspective of the users at the center of our design thinking process.”-*ADHSB09*

“The program effectively blends theoretical knowledge with practical applications, *incorporates* industry-relevant case studies, and encourages collaborative learning. It equips participants with a holistic understanding of digital health, making it valuable for anyone looking to navigate this dynamic and crucial field.”-*ADHSB27*

“The bootcamp pretty much covers all important facets of Digital Health, and the addition of world-class mentors makes it awesome.”-*ADHSN06*

### On exposing participants to hands-on knowledge and skills for practical application (objective 3)

“The knowledge learnt during the bootcamp has exposed me to the application of several technologies to health. Together with my team member, I was able to come up with a solution to a problem in health, and I hope to implement the knowledge gathered to execute amazing projects in the future.”-*ADHSB44*

“I leveraged my market research skills acquired during the bootcamp to conduct thorough analyses for various projects. This involved gathering and interpreting relevant data, identifying market trends, and assessing consumer needs. Through strategic application of these research insights, I contributed to informed decision-making, enabling the projects to align more effectively with market demands and opportunities. Also, I implemented key leadership skills acquired during the bootcamp to enhance team collaboration and foster a more cohesive work environment. By applying effective communication, delegation, and motivational strategies, I successfully contributed to team formation, resulting in improved productivity and a positive work culture.”-*ADHSB14*

### On fostering collaboration among students in different universities across Africa (objective 4)

“It’s a great opportunity for professionals to explore the intersection between health and technology. And have the capacity to leverage. Moreover, connecting from different people across Africa is major advantage for future collaboration for the collective advantage of the professionals and the work they produce.”-ADHSB47

“Yes, I would definitely recommend this bootcamp to anyone interested in learning more about digital health and starting a career in this field. It was a great experience that taught me a lot about the field and helped me prepare for a career in digital health. I really liked that the bootcamp focused on real-world applications and hands-on learning. This helped me to understand how digital health is used in the real world and gave me the skills I need to start my own projects. Besides the great curriculum and instructors, I also liked the bootcamp’s focus on collaboration and networking. I got to work with a diverse group of people from around Africa, and we learned a lot from each other. We also had the chance to meet professionals in the digital health industry, which was very helpful.”-ADHSB25

### On identifying healthcare challenges in their community and building uniquely tailored tech-driven solutions (objective 5)

“I was able to recognize a health problem that wasn’t being addressed with the use of digital health and implement a market analysis, prototyping using technologies I’ve never used before successfully. To develop a solution for our innovation challenge. And I will continue with the solution with my colleague beyond this boot camp. I learned interesting ways I can develop my career in sequencing, which is an area I’m most interested in. As a student, I’m always preparing presentations, and I believe I’ve received masterclass training on how to make them more engaging. This boot camp has positively impacted my overall knowledge.”-ADHSB47

“I am currently applying design thinking in the development of a mobile health application that caters to the management of people living with non-communicable diseases, specifically hypertension and diabetes”-ADHSN11

“I used my user research knowledge learnt from the program during our innovation challenge. I also applied the Prototyping skill I learnt to build a prototype for our Mindconnect Product.”-ADHSB36

### Feedback that covers at least three objectives

“I would recommend the digital health bootcamp for several reasons. The curriculum is robust, covering not only foundational concepts but also emerging technologies that are crucial to the evolving digital health landscape. The instructors bring a wealth of experience to the table, providing practical insights and real-world applications. Also, the potential for networking opportunities and industry partnerships can greatly enhance your learning experience, providing valuable connections and insights for your future endeavors in digital health. Overall, the program’s comprehensive and interdisciplinary approach, coupled with its flexibility and continuous learning opportunities, makes it a solid recommendation for anyone looking to excel in the dynamic field of digital health.”-ADHSB14

“Yes, I would definitely recommend this bootcamp to anyone interested in learning more about digital health and starting a career in this field. It was a great experience that taught me a lot about the field and helped me prepare for a career in digital health. I really liked that the bootcamp focused on real-world applications and hands-on learning. This helped me to understand how digital health is used in the real world and gave me the skills I need to start my own projects. Besides the great curriculum and instructors, I also liked the bootcamp’s focus on collaboration and networking. I got to work with a diverse group of people from around Africa, and we learned a lot from each other. We also had the chance to meet professionals in the digital health industry, which was very helpful.”-ADHSB25

“Yes, I would recommend this bootcamp to others interested in digital health. The comprehensive curriculum, expert-led sessions, and practical insights have equipped me with valuable knowledge and skills. The interactive nature fosters meaningful learning, making it an excellent choice for anyone passionate about making a positive impact in digital health.”-ADHSN01

### Recommendations and expectations for future bootcamps

“Exploring more emerging technologies like AI in healthcare, ethical considerations in digital health, and telemedicine advancements would provide a comprehensive view.”-ADHSN01

“More on fragmentation and interoperability, Global goods, and in-depth about regulations and policies that govern digital health in Africa.”-ADHSB40

“How to be digital health champions as individuals around people opposed to the idea.”-ADHSB37

“A more similar event could be held on more advanced topics for the graduate cohort.”-ADHSB01

“I encourage more interactive sessions to foster engagement and facilitate a deeper understanding of complex digital health concepts.”-ADHSB14

“The session on Entrepreneurship/Financing in Digital Health should have been taken. It is quite an important knowledge area for digital health start-ups, especially in the face of the current harsh economic weather in Africa.”-ADHSB09

### Future opportunities

The inaugural bootcamp offers an opportunity to expand its reach and impact by scaling up the program to include more participants across Africa through the student networks being developed at various African universities. It creates a chance to strengthen relationships with educational institutions, government agencies, and industry stakeholders, thereby boosting the effectiveness of future digital health education initiatives. Due to the bootcamp's success, the Africa Canada AI and Data Consortium aims to collaborate with DHA to establish an AI in Healthcare Club within our student network and to run an AI in Healthcare Bootcamp in 2024. Furthermore, the bootcamp allows DHA to build a pool of experts they can draw upon for other programs and initiatives. They also maintain an alumni page for all bootcamp participants to track their progress and continue supporting their interest in health innovations.

## Discussion

In this bootcamp, the participants were predominantly aged between 18 and 35 (69.8%), suggesting that the bootcamp targeted younger individuals who are mostly considered early career professionals and the most active demographic in many programs. This age group reflects the growing youth demographics of the continent, with their particular interest in utilizing digital health to develop innovative solutions, given the emergence and rapid growth of the digital health sector. Young people are often more adaptable and eager to learn new skills, making them more suited to maximize the potential of digital transformation (19). Moreover, the study had a relatively equal representation of both male and female participants, with a gender breakdown of 49% male and 50% female. This relatively balanced representation ensures that the findings are not biased towards one gender, allowing for a comprehensive understanding of how both genders perceive and benefit from digital health education. This underlines the inclusive approach to empowering both genders in digital health skills, emphasizing that digital health education and career opportunities should be accessible and appealing to a broad audience, which aligns with Romi's study (20). Although this is in contrast to an Ethiopian IT internship program, which directly addressed skilled workforce gaps in digital health, it reported only 24% female participation due to a limited pipeline of women in IT-related fields, whereas our bootcamp achieved relatively gender parity. This contrast underscores the importance of intentional recruitment strategies in digital health education, as gender imbalances in training programs can translate into inequities in the future workforce and limit the inclusivity of digital health innovations (21).

The participants were distributed across various years of study, from the 1st year to the 6th year, with a balanced distribution across different years. This suggests that the bootcamp appealed to students at various stages of their academic journey. The higher representation of 4th-year students (20.8%) may indicate a greater interest and career clarity in digital health among those approaching the final stages of their education, potentially as they prepare for their professional careers. In contrast, the extended hackathon model (22) reported that graduate students -those enrolled in Master's, PhD, or MBA programs- comprised the largest demographic (33.5%) across eight events. This difference in participant profiles highlights how program design, such as eligibility criteria, including the target group and outreach strategies, can influence participant engagement. While our bootcamp offered accessible entry points for undergraduates, the extended hackathon's emphasis on advanced interdisciplinary collaboration and prototyping might have been more appealing to graduate-level participants with specialized interests.

The participants came from 13 different African countries, reflecting a diverse regional cohort. The majority of participants were from Nigeria, representing a significant portion of the total participants. This diverse representation of participants from various African countries suggests that the bootcamp successfully attracted a wide range of students from different cultures and geographic settings, with varied backgrounds and experiences. The regional nature of the cohort, with a notable majority from Nigeria, may have enriched the learning experience by providing different perspectives on digital health. This is consistent with a 48-hour virtual public health hackathon that also included 13 different African countries, including Nigeria, Ghana, Kenya, Uganda, Cameroon, Tanzania, and Sudan. Nigeria again represented the largest share of participants (49 entries) (23), which might suggest its central role in youth-led health innovation, internet access for youths and students across the country, and their growing interests in digital health education. This rich geographic diversity in both initiatives is significant because it validates the pan-African relevance of digital health education and innovation. It enhances solution contextualization, as participants bring localized insights into public health challenges, and it promotes regional equity, ensuring that digital health capacity-building is not limited to a single country or institution.

The study measured participants' familiarity with digital health concepts before the bootcamp and their level of improvement after completing the bootcamp. The level of improvement observed among participants was remarkable. The strong correlation between initial familiarity and subsequent improvement highlights the bootcamp's effectiveness in empowering participants and enhancing their knowledge. The majority of participants showed exceptional and significant improvement (80.94%, 82.93%), indicating that the bootcamp successfully built upon their existing knowledge.

A significant portion of participants also demonstrated substantial improvements in knowledge of digital health concepts across the seven modules that comprised the ADHSB curriculum, with 80.49% showing moderate improvement and 36.59% showing slight improvement, thus elevating their understanding to a more advanced level. Even participants with little to no prior knowledge experienced varying levels of improvement, underscoring the bootcamp's ability to introduce new concepts effectively. Overall, the boot camp proved highly effective in equipping participants with essential digital health skills, hence empowering them with the requisite skills to tackle health challenges in Africa. Our findings are consistent with (23), who documented a public health hackathon for African medical students that combined training, mentorship, and digital health prototyping. Their results showed that participants -regardless of prior experience- gained confidence and developed impactful innovations, echoing our observation that structured bootcamp formats can effectively elevate digital health competencies across diverse learner profiles.

In addition, the findings are also consistent with the broader context outlined by Wong et al. (19), which highlights the significant potential of digital health to enhance healthcare outcomes and promote global health equity. However, it underscores that effective implementation and responsible use of digital health tools require a health workforce with sufficient knowledge and skills to navigate digital transformations in health. Wong and colleagues highlighted the importance of capacity building and the development of digital literacy to ensure that digital health tools are used correctly and competently in practice. Our study supports this assertion, as participants were empowered, as demonstrated by the significant improvement in their familiarity with digital health concepts following the bootcamp. This improvement underscores the structured approach to implementing the bootcamp, which includes the effectiveness of practical, hands-on learning experiences in enhancing participants' familiarity with digital health concepts and may serve as a foundation for building future competencies in digital health. While our bootcamp successfully improved participants' familiarity with digital health concepts, a review article emphasized that sustainability requires broader organizational and community-level factors, including stakeholder commitment and integration into health systems (24).

Furthermore, the bootcamp was specifically effective in enhancing participants' knowledge of product management and design thinking within digital health, which may be due to their ability to grasp and apply the concepts taught in these modules effectively in their project. These findings resonate with a previous study that reported on a hackathon embedded in a biosecurity and pandemic resilience course. Their study demonstrated that design thinking-based hackathon challenges fostered interdisciplinary collaboration and generated 52 potentially implementable solutions to COVID-19, including prototypes that mirrored industry responses such as Bluetooth contact-tracing apps. While hackathons excel at driving innovation outputs through diverse teamwork and collaboration, bootcamps can also provide sustained, domain-specific capacity building in digital health, highlighting the importance of both initiatives and their complementary roles, strengths, and the invaluable nature of both approaches (25).

In addition, a previous study conducted on international university students in 2024 (26) highlights the necessity of addressing various categories of digital skills—common, work, and advanced- among university students to ensure their preparedness for everyday life and the workplace. The study reports that the common digital skills gap ranges from 10% to 48%, the work digital skills gap ranges from 14% to 40%, and the advanced digital skills gap ranges from 8% to 54%, with a significant portion of advanced digital skills gaps exceeding 30%. In comparison, our bootcamp aimed to enhance participants' familiarity with digital health concepts across different modules, addressing various levels of digital skills. This aligns with (22), who reported a 114.1% increase in self-assessed knowledge across 10 domains of medical innovation following a structured, extended hackathon model. Their curriculum included design thinking, app/web prototyping, and business model development, domains that closely mirror the ADHSB's highest-performing modules. Notably, their study found that undergraduate participants showed the greatest improvement, particularly in the Stanford 2017 extended hackathon, reinforcing our observation that early-career learners benefit most from immersive, interdisciplinary formats. While their hackathons spanned multiple international sites, our bootcamp specifically addressed digital health skill gaps in African contexts, offering targeted training across foundational, work-related, and advanced digital competencies. Unlike (22), which had a broader medical innovation focus, the ADHSB bootcamp concentrated on digital health systems, processes, and tools, offering a more domain-specific approach to capacity building.

The bootcamp and the study (19) highlight the critical importance of comprehensive digital health education. By addressing various levels of digital skills and providing practical, hands-on learning experiences, educational programs can effectively empower and prepare the next generation of health professionals to navigate and drive digital transformations in healthcare (27).

The bootcamp successfully achieved its objectives. These achievements demonstrate that structured digital health literacy and educational interventions can bridge practice gaps and build both knowledge and confidence among early-career learners, laying the foundation for a digitally competent health workforce in Africa. The increase in knowledge observed across our modules has important implications for workforce outcomes. As participants gain familiarity with digital health concepts, they are better positioned to contribute to improved data use, patient care, and context-specific innovation within their communities.

Nevertheless, sustainability remains a critical consideration. Beyond short-term knowledge gains, long-term impact requires founders, digital health experts, and ministries of health to champion these projects, ensure continuity, and integrate them into national strategies. As highlighted in a review on barriers and facilitators for the sustainability of digital health interventions in low and middle-income countries (24), sustainability depends on stakeholder commitment, infrastructure investment, and workforce retention. The ministries of health in each country on the continent should therefore view bootcamps not only as training exercises but as strategic investments in human capital that can drive digital transformation across health systems.

Regarding the policy implications of our study, we suggest that governments and institutions support the integration of digital health education into medical and health sciences curricula to ensure early exposure for students (28). Also, partnerships with industry and incubators should be institutionalized to bridge the gap between training and employment, thereby reducing workforce shortages. Furthermore, ministries of health should adopt policies that encourage sustained mentorship and funding for youth-led digital health projects, ensuring that innovations generated in bootcamps and hackathons do not remain prototypes but evolve into scalable solutions, aligning educational initiatives with national digital health strategies (29). Through this, Africa can build a resilient workforce capable of sustaining digital health interventions and advancing health equity across the continent.

Similarly, the bootcamp significantly empowered participants with the requisite knowledge needed to innovate on contextual health issues across the continent and also improved their understanding of digital health concepts and skills. Therefore, if similar opportunities are intentionally extended to Lusophone and Francophone countries, this will reduce the widened digital health workforce gaps in these regions (28). In this light, a regionally inclusive approach would support African-wide digital health literacy and national strategies to achieve digital health, thereby contributing to achieving universal health coverage across the continent (28).

Expanding ADHSB-type initiatives to Lusophone and Francophone presents an opportunity for strategic partnerships with Francophone and Lusophone universities, regional bodies and youth networks. This opportunity can be leveraged to extend and establish ADHSN at each of these universities, which could foster sustained student engagement, knowledge exchange, a sustained mentorship pipeline, improved collaboration, and a larger innovation ecosystem of student networks with the same mission and vision statement (30).

Limitations of this study included the limited number of participants, which cannot be considered representative of the entire African continent. Additionally, while the ideation and innovation phases of the bootcamp were engaging, they did not include a longitudinal follow-up to monitor whether the proposed innovations were eventually implemented, scaled, or sustained, making it challenging to evaluate the long-term effectiveness and real-world impact of the projects. The time zone differences really affected participation, contributing to dropouts and truancies in attending sessions.

Moreover, while the majority of participants in this cohort are from anglophone countries with students from Nigeria accounting for the largest proportion of the total participants, the linguistic imbalance presents dire implications for youth empowerment in digital health skills across Lusophone and Francophone countries with similar health system challenges and barriers to digital health adoption. Therefore, opportunities for such initiatives continue to be limited in these settings. The delivery of ADHSB curriculum, mentorship and workshop sessions, pitch presentations, and judges was primarily conducted in English, which may unintentionally exclude talented students from French and Portuguese-speaking regions on the continent. This suggests a need for a multilingual program design, including translated learning materials, bilingual mentorship and interpretation support for French and Portuguese speakers to ensure equitable participation across Africa.

The strengths of the study include the provision of free days for workshops, during which participants were not required to attend training sessions but were instead allowed to focus on developing and refining their ideas, preparing their pitch decks, and practicing demo pitches. The assignment of mentors to each group allowed participants to interact and engage with them, gaining clarity and direction on their ideas during the ideation phase and the final project presentation. It is believed that this helped refine their ideas, and the free days helped reduce the stress of balancing school schedules with the bootcamp. The varied time zones were considered during the bootcamp, and adjustments were made to accommodate differences when necessary. Additionally, the prize money incentive motivates participants and fosters a sense of dedication to learning and completing the program.

Future research is necessary to assess the long-term effects of such initiatives, including how participants apply their skills in real-world contexts and whether the innovations developed are implemented or scaled. Moreover, additional studies can investigate effective strategies for scaling successful interventions and overcoming barriers encountered during the implementation phase.

## Conclusion

The ADHSB'23 effectively empowered participants with essential digital health skills and knowledge. Participants demonstrated significant improvements in their familiarity with digital health concepts, particularly in modules such as “Product Management in Digital Health” and “Design Thinking in Digital Health.” The improvement in these skills could be the result of direct support and application in problem-solving and innovation during their ideation phase. In future bootcamps, these areas need to be prioritized.

The bootcamp effectively builds the participants' knowledge in digital health concepts and literacy to navigate and drive digital transformations in healthcare, contributing to improved healthcare outcomes and global health equity. Even participants with no prior knowledge showed significant improvement across modules. Moreso, all the proposed targets were met and exceeded, yielding a significant number of positive outcomes. The bootcamp also demonstrated that young people can quickly acquire digital health skills if exposed to the right information, resources and environment to achieve that, especially a structured mentorship approach, practical sessions, live demos, and collaborative projects. Therefore, it is crucial to draw key lessons from this cohort and strive to expand the program to reach more young people in other regions of Africa, as this will ensure an empowered young people on the continent.

The program attracted participants from 13 African countries across multiple fields, enriching the learning process. The diversity created opportunities for all participants to engage in cross-cultural learning and approaches, broader problem perspectives, and a contextually relevant approach to healthcare solutions. Also, the opportunity for these participants to learn from subject matter and industry experts further enriched the experience as this improved teamwork, confidence among students to develop and pitch solutions and also helped them navigate technical and non-technical concepts. In addition, the opportunity to expose them to practical projects proved that young people and students can produce fundable and scalable digital health innovations when given the proper support.

Furthermore, this bootcamp created an opportunity to expand the African Digital Health Student Network (ADHSN) to the ADHSB participants' universities. This expansion will further create strategic partnerships with those universities for future virtual or in-person programs or bootcamps, leading to long-term ecosystem building. The bootcamp generated various outcomes, including skill acquisition, novel ideas, and solution presentations, it is vital to showcase participants innovations and creativity to stakeholders in the field such as digital health organizations, policymakers, academia, media, government, universities, investors and the general public to raise awareness and recognition of the program and participants as this will increase opportunities for implementation and scaling.

Additionally, participants should receive ongoing follow-up and support, such as mentorship, internships, funding, or networking opportunities, which will help them achieve their goals and contribute to the integration of the digital health industry into healthcare sectors. This will also help to sustain alumni engagement to maintain momentum beyond the bootcamp. Generally, the bootcamp proved to be an effective tool for enhancing digital health literacy, education, collaboration, innovation, and addressing practice gaps among young African people and students.

## Data Availability

The raw data supporting the conclusions of this article will be made available by the authors, without undue reservation.
