# Supplementary material for: The African digital health student bootcamp: bridging education, workforce, and practice gaps for healthcare innovation in Sub-Saharan Africa
Source: Front Digit Health. 2026 Feb 4;8:1728386. doi: 10.3389/fdgth.2026.1728386 (PMC12914563; doi:10.3389/fdgth.2026.1728386)
Supplement: Supplementary file 1 [file Datasheet1.pdf]

DIGITAL HEALTH AFRICA

October 2023

AFRICAN DIGITAL HEALTH STUDENT BOOTCAMP CALENDAR SEPTEMBER 2023 - DECEMBER 2023

WEEK	MONDAY	TUESDAY	WEDNESDAY	THURSDAY	FRIDAY	SATURDAY	SUNDAY	TOPIC
1	2	3	4	5	6	7		BASIC INTRODUCTION TO DIGITAL HEALTH
			MENTORSHIP SESSION			Introduction to Digital Healthcare		
			Leadership and Management in Digital Health			Overview of digital healthcare innovations and their impact on the healthcare industry		
2	9	10	11	12	13	14		Healthcare Data And Analytics
			MENTORSHIP SESSION		Introduction to Healthcare Data	Data-Driven Decision-Making in Healthcare		
			My health , My Data - Health Data Governance		Definition and key concepts in healthcare data analytics	Data Visualization and Reporting		
3	16	17	18	19	20	21		Change Management in Digital Health
			MENTORSHIP SESSION					
			Becoming a digital health advocate in Africa			Gender Equality, Diversity and Inclusion in digital health		
4	23	24	25	26	27	28	29TH	Product Management In Digital Health
			MENTORSHIP SESSION			Introduction to Product Management in Digital Health	Individual Design Challenge	
			Collaboration and Networking skills			Identifying Digital Health Opportunities and Market Analysis		

November 2023

AFRICAN DIGITAL HEALTH STUDENT BOOTCAMP CALENDAR SEPTEMBER 2023 - NOVEMBER 2023

WEEK	WEDNESDAY	THURSDAY	FRIDAY	SATURDAY	SUNDAY	TOPIC	
5	1	2	3	4	5	DESIGN THINKING IN DH/CHIP	5
	MENTORSHIP SESSION	Final Team Design Challenge Framing	Introduction to Design Thinking in Digital Health	Module 3: Defining the Problem in Digital Health	Persona Template		
				Module 4: Ideation and Creativity in Digital Health Solutions			
	Case study on: Implementing and Scaling Digital Health Innovations			Module 5: Prototyping Digital Health Solutions			
				Module 6: Testing and Iterating Digital Health Prototypes			

WEEK	MONDAY	TUESDAY	WEDNESDAY	THURSDAY	FRIDAY	SATURDAY	SUNDAY	TOPIC	
6	6	7	8	9	10	11	12	EMERGING DH TECH	6
		Market Research Presentation	DEMO FROM GOOGLE HEALTH		Blockchain Technology in Healthcare	Artificial Intelligence (AI) in Healthcare	Interview Guide		
						Genomics and Personalized Medicine			
							Empathy Map		
7	13	14	15	16	17	18	19	HEALTHCARE ENTREPRENEURSHIP	7
		Define/Problem Framing Presentation	Digital Health Startup Founders/Fireside Chat	How Might We Q's		Check in session/ Happy Hour	Prototyping and Storyboarding		
		Insight Statements			Pitch Template				
8	20	21	22	23	24	25		COMMUNITY HEALTH INNOVATION PROJECT	8
	Solution Catalogue				MOCK PITCH (FRIDAY EVENING)	DEMO DAY (SATURDAY EVENING)			
9	27	28	29TH					GRADUATION	

FINAL EVENT/CLOSE OUT (SATURDAY EVENING)

SATURDAY	SUNDAY	MONDAY	TUESDAY	WEDNESDAY	THURSDAY	FRIDAY
8	9	10	11	12	13	14
6-Step Innovation	Individual Design Challenge	Final Team Design Challenge Framing		Market Research Presentation	Define/Problem Framing Presentation	
Video 1 and 2				Interview Guide	Insight Statements	
HCD Welcome Survey		Persona Template		Empathy Map	How Might We Q's	

SATURDAY	SUNDAY	MONDAY	TUESDAY	WEDNESDAY	THURSDAY	FRIDAY
15	16	17	18	19	20	21
Team Brainstorm	*		Prototyping and Storyboarding	Solution Catalogue	Pitch Presentation	Team member and leader evaluation
Idea Sketches			Pitch Template		**	Challenge Survey
