# Supplementary material for: The African digital health student bootcamp: bridging education, workforce, and practice gaps for healthcare innovation in Sub-Saharan Africa
Source: Front Digit Health. 2026 Feb 4;8:1728386. doi: 10.3389/fdgth.2026.1728386 (PMC12914563; doi:10.3389/fdgth.2026.1728386)
Supplement: Supplementary file 2 [file Datasheet2.pdf]

ADHSB 23 Post Survey

Dear Participant,

Thank you for being a part of our Digital Health Bootcamp. Your feedback is invaluable to us as we strive to improve and tailor our future programs to better meet your needs. This survey aims to gather your insights and experiences regarding the bootcamp sessions and content.

Your responses will remain anonymous and will be used solely for the purpose of program evaluation and enhancement. This survey should take approximately 15 minutes to complete.

Please take a moment to reflect on your experience during the bootcamp and provide us with your honest feedback. Your input will help us in shaping future programs to better serve our participants.

Thank you for your time and contribution. Let's begin!

* Indicates required question

Email *

☐ Record my email address with my response

Age *

Pre-fill responses, then click "Get link"

Gender *

- Male
- Female

Current level of study *

- Undergraduate
- Postgraduate
- Other:

ADHSB 23 ID Number *

Your answer

Overall Experience

On a scale of 1 to 5 how satisfied were you with the Digital Health Bootcamp? *

	1	2	3	4	5	
Less Satisfied	○	○	○	○	○	Extremely Satisfied

Did the bootcamp meet your expectations? *

- Yes

Pre-fill responses, then click "Get link"

Learning Outcomes

Which topics or modules did you find most beneficial?
Please rate your level of improvement in understanding with the following digital health concepts on a scale of 1 to 5 where;

1 No Improvement
2: Slight Improvement
3: Moderate Improvement
4: Significant Improvement
5: Exceptional Improvement

Module 1:Foundations of Digital Health *

	1	2	3	4	5
Electronic Health Records	○	○	○	○	○
Mobile Health(m-health)	○	○	○	○	○
Health Information Exchange	○	○	○	○	○
AI/ML	○	○	○	○	○

Pre-fill responses, then click "Get link"

Module 2: Healthcare Data and Analytics *

	1	2	3	4	5
Healthcare Data	○	○	○	○	○
Healthcare Analytics	○	○	○	○	○
Clinical Decision Support	○	○	○	○	○
Operations management efficiency	○	○	○	○	○

Module 3: Change Management in Digital Health *

	1	2	3	4	5
Process Improvement Initiatives	○	○	○	○	○
Quality Improvement Initiatives	○	○	○	○	○
Cultural transformation	○	○	○	○	○
Stakeholder Engagement	○	○	○	○	○
Change	○	○	○	○	○

Pre-fill responses, then click "Get link"

Module 4: Product Management in Digital Health *

	1	2	3	4	5
Market Research Analysis	○	○	○	○	○
Product strategy and Planning	○	○	○	○	○
Requirements, gathering and prioritization	○	○	○	○	○
User Experience Design	○	○	○	○	○
Development and Release Management	○	○	○	○	○
Performance monitoring and optimization	○	○	○	○	○

Pre-fill responses, then click "Get link"

Module 5: Design Thinking in Digital Health *

	1	2	3	4	5
Ideation	○	○	○	○	○
Prototyping	○	○	○	○	○
Testing	○	○	○	○	○
Implementation	○	○	○	○	○
Evaluation and iteration	○	○	○	○	○
Human Centered Design	○	○	○	○	○

Pre-fill responses, then click "Get link"

Module 6: Digital Health Entrepreneurship *

	1	2	3	4	5
Identifying Healthcare Challenges	○	○	○	○	○
Developing Digital Health solutions	○	○	○	○	○
Collaboration	○	○	○	○	○
Regulatory Compliance	○	○	○	○	○
Funding and business models	○	○	○	○	○
Scalability and Growth	○	○	○	○	○
Market research and Validation	○	○	○	○	○

Pre-fill responses, then click "Get link"

Module 7: Emerging Technologies in Digital Health *

	1	2	3	4	5
Artificial intelligence	○	○	○	○	○
Internet of Things	○	○	○	○	○
Telemedicine	○	○	○	○	○
Precision Medicine and Genomics	○	○	○	○	○

Practical Application

Have you implemented any concepts learned during the bootcamp in your work or personal life? *

○ Yes

○ No

If yes, please describe how you applied the knowledge gained.

Your answer

Instructor Evaluation

Pre-fill responses, then click "Get link"

On a scale of 1 to 10 Rate the effectiveness of the instructors in delivering the course content *
(1represents less effective and 5 most effective)

	1	2	3	4	5
Module 1	○	○	○	○	○
Module 2	○	○	○	○	○
Module 3	○	○	○	○	○
Module 4	○	○	○	○	○
Module 5	○	○	○	○	○
Module 6	○	○	○	○	○
Module 7	○	○	○	○	○

Any comments or suggestions regarding the instructors? *

Your answer

Future Improvement

How can we enhance future bootcamps on digital health? *

Your answer

Pre-fill responses, then click "Get link"

What additional topics or areas would you have liked to see covered in the bootcamp? *

Your answer

Recommendations

Would you recommend this bootcamp to others interested in digital health? *

- Yes
- No

Please share why or why not. *

Your answer

General Feedback

Any other comments or feedback you would like to provide? *

Your answer

Visual Representation

Dear Participants,We're excited to compile a collective visual representation of our cohort's journey through the Digital Health Bootcamp! To create a vibrant collage or showcase on our website, we invite each participant to contribute an image that encapsulates your experience, insights, or inspiration gained from the bootcamp.

Submission Guidelines

Pre-fill responses, then click "Get link"

Ensure the image is high-resolution and has relevance to the digital health domain.

Upload your image to a cloud storage platform (Google Drive, Dropbox, etc.) and generate a shareable link.

How to Submit:

Upload your image to the provided cloud storage platform.

Generate a shareable link to the image file.

Complete the submission form using the link below

Important Note:By submitting your image, you grant us permission to use it in the cohort collage or on the bootcamp website. Your contribution will be a valuable addition to our shared experience and will inspire fellow participants. Thank you for sharing your creativity and insights with us!

Link to Image *

Your answer

Get link

Never submit passwords through Google Forms.

This content is neither created nor endorsed by Google. - Contact form owner - Terms of Service - Privacy Policy

Does this form look suspicious? Report

Google Forms

Pre-fill responses, then click "Get link"

Pre-fill responses, then click "Get link"
