# Supplementary material for: The African digital health student bootcamp: bridging education, workforce, and practice gaps for healthcare innovation in Sub-Saharan Africa
Source: Front Digit Health. 2026 Feb 4;8:1728386. doi: 10.3389/fdgth.2026.1728386 (PMC12914563; doi:10.3389/fdgth.2026.1728386)
Supplement: Supplementary file 3 [file Datasheet3.pdf]

ADHSB 23 Pre Survey

Unlocking a Great Experience

Our program is all about growth, innovation and learning. By taking a few moments to complete this pre-survey, you're helping us unlock the full potential of your experience.

* Indicates required question

Email *

Cannot pre-fill email

Age *

Your answer

Gender *

- Male
- Female
- Prefer not to say
- Other:

Pre-fill responses, then click "Get link"

Educational level *

Choose

Current year of study

- Year 1
- Year 2
- Year 3
- Year 4
- Year 5
- Year 6

Experience with the Concepts covered in ADHSB' 23

Please rate your level of familiarity or experience with the following digital health concepts on a scale of 1 to 5 where;

1 Very unfamiliar
2 Unfamiliar
3 Somewhat familiar
4 Familiar
5 Very Familiar

Pre-fill responses, then click "Get link"

Module 1:Foundations of Digital Health *

	1	2	3	4	5
Electronic Health Records	○	○	○	○	○
Telehealth	○	○	○	○	○
Mobile Health(m-health)	○	○	○	○	○
Health Information E	○	○	○	○	○
AI/ML	○	○	○	○	○

Pre-fill responses, then click "Get link"

Module 2: Healthcare Data and Analytics *

	1	2	3	4	5
Healthcare Data	○	○	○	○	○
Healthcare Analytics	○	○	○	○	○
Clinical Decision Support	○	○	○	○	○
Population Health management	○	○	○	○	○
Operations management efficiency	○	○	○	○	○

Pre-fill responses, then click "Get link"

Module 3: Change Management in Digital Health *

	1	2	3	4	5
Process Improvement Initiatives	○	○	○	○	○
Quality Improvement Initiatives	○	○	○	○	○
Cultural Transformation	○	○	○	○	○
Stakeholder Engagement	○	○	○	○	○
Change management training	○	○	○	○	○

Pre-fill responses, then click "Get link"

Module 4: Product Management in Digits Health *

	1	2	3	4	5
Market Research and Analysis	○	○	○	○	○
Product Strategy and Planning	○	○	○	○	○
Requirements,Gathering and Prioritization	○	○	○	○	○
(UX) User Experience Design	○	○	○	○	○
Development & Release Management	○	○	○	○	○
Agile Methodology	○	○	○	○	○
Performance monitoring and Optimization	○	○	○	○	○
Competitive Analysis	○	○	○	○	○

Pre-fill responses, then click "Get link"

Module 5: Design Thinking in Digital Health *

	1	2	3	4	5
Ideation	○	○	○	○	○
Prototyping	○	○	○	○	○
Testing	○	○	○	○	○
Implementation	○	○	○	○	○
Evaluation and Iteration	○	○	○	○	○
Human centered design	○	○	○	○	○

Pre-fill responses, then click "Get link"

Module 6: Digital Health Entrepreneurship *

	1	2	3	4	5
Identifying Healthcare Challenges	○	○	○	○	○
Developing Digital Health Solutions	○	○	○	○	○
Collaboration with proffesionals	○	○	○	○	○
Regulatory Compliance	○	○	○	○	○
Funding and Business Models	○	○	○	○	○
Scalability and Growth	○	○	○	○	○
Market Research and Validation	○	○	○	○	○

Pre-fill responses, then click "Get link"

Module 7: Emerging Technologies in Digital Health *

	1	2	3	4	5
Artificial Intelligence(AI)	○	○	○	○	○
Internet of Things(IOT)	○	○	○	○	○
Telemedicine	○	○	○	○	○
AR/VR	○	○	○	○	○
Precision Medicine and Genomics	○	○	○	○	○
Blockchain	○	○	○	○	○

Pre-fill responses, then click "Get link"

Which of the following digital health concepts are you most looking forward to learning more about as it relates to your professional field *

Choose 5 from the list below

- [ ] Electronic Health Records
- [ ] Telehealth
- [ ] AI and ML
- [ ] Health Data Exchange
- [ ] Healthcare Analytics
- [ ] M-Health(Mobile health)
- [ ] UX Design
- [ ] Clinical Decision Support
- [ ] Operational Efficiency Management
- [ ] Human Centered
- [ ] Population Health Management
- [ ] Agile Methodology
- [ ] Prototyping
- [ ] Ideation
- [ ] Competitive Analysis
- [ ] Market Research and Analysis
- [ ] Stakeholder Engagement
- [ ] Cultural Transformation
- [ ] Quality Improvement Initiatives

Pre-fill responses, then click "Get link"

☐ Other:

Get link

Never submit passwords through Google Forms.

This content is neither created nor endorsed by Google. - Contact form owner - Terms of Service - Privacy Policy

Does this form look suspicious? Report

Google Forms

Pre-fill responses, then click "Get link"

Pre-fill responses, then click "Get link"
